# Anaplastic Sarcoma of the Kidney

**DOI:** 10.1100/tsw.2009.15

**Published:** 2009-02-15

**Authors:** Apostolos P. Labanaris, Vahudin Zugor, Robert Smiszek, Reinhold Nützel, Reinhard Kühn

**Affiliations:** ^1^ Department of Urology, Martha Maria Medical Center, Nuremberg, Germany; ^2^ Department of Urology, Salzgitter Medical Center, Salzgitter, Germany

**Keywords:** anaplastic sarcoma of the kidney, anaplastic Wilms’ tumor, diagnosis, therapy

## Abstract

We present a case of an extremely rare and relatively new tumor entity of the kidney, the anaplastic sarcoma. Although of unknown origin and pathogenesis, treating such a tumor as if it was anaplastic Wilms' tumor seems to be the only therapeutic solution at the present time. Newer immunohistochemical staining and molecular probes should be applied to this neoplasm in order for us to understand it nature and maximize therapy.

## INTRODUCTION

Wilms' tumor is the most common primary malignant renal tumor of childhood, and represents approximately 6% of all childhood cancers and about 1% of all adult renal tumors in Europe and the U.S. Because it can appear with a variety of morphologic features, it can sometimes obscure other clinicopathologic entities of the kidney. We present a case of a patient that was initially diagnosed as having anaplastic Wilms' tumor, but further histopathological and immunohistochemical examinations revealed an extremely rare and relatively new tumor entity of the kidney, the anaplastic sarcoma, first described by Vujanić et al.[1].

## CASE

In March 2006, a 24-year-old woman presented to our department complaining of right flank pain for the last 2 weeks. Weight loss and fatigue were also observed. Physical examination revealed a palpable, smooth abdominal mass. Laboratory findings exhibited a slight anemia and urinary sediment revealed an insignificant amount of white blood cells. Ultrasound revealed a heterogeneous mass within the right kidney, with a solid lesion and a central area of increased reflectivity and, in some parts, with an ecogenicity similar to that of the renal parenchyma measuring about 22 × 10 cm. MRI of the abdomen confirmed the findings and additionally excluded lymph node involvement, extrarenal tumor extension into the perirenal fat or adjacent structures, and vena cava involvement (Fig. 1). CT of the thorax was negative for lung metastasis. The preoperative diagnosis made was that of Wilms' tumor or renal cell carcinoma. Radical nephrectomy with regional lymphadenectomy was performed. A complete resection with negative surgical margins was achieved and no significant intraoperative complications were noted.

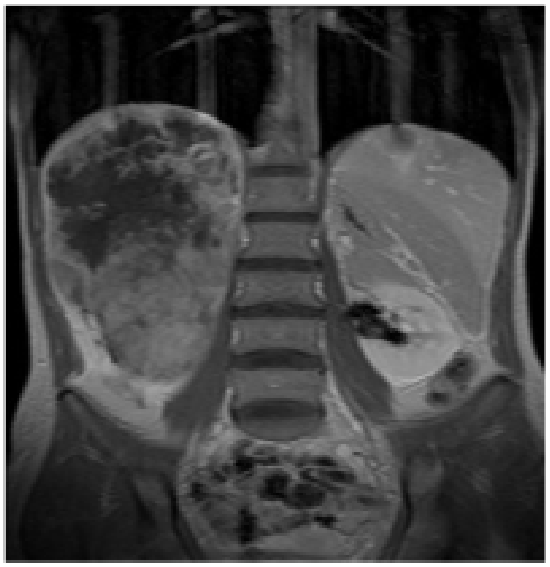

**FIGURE 1.** MRI of a heterogeneous mass within the right kidney.

The size of the tumor retrieved through the nephrectomy specimen was about 22 × 12 cm and revealed a distinct cystic component. At first, a histopathological diagnosis of an unusual anaplastic Wilms' tumor Stage II, without lymph node involvement was made. Because of its unusual nature, a further histopathlogical examination was performed. The histologic features exhibited small primitive mesenchymal cells coexisting with a spindle cell component (Fig. 2). Anaplastic nuclear changes exhibiting atypical protean multinucleated cells that contained large anomalous hyperchromatic nuclei and that were dispersed throughout the tumor islands of hyaline cartilage were also evident. Other features included the presence of multicompartmented cysts separated by thin fibrous septa, foci of necrosis, as well as single-cell necrosis in the solid tumor area. Characteristics consistent with Wilms' tumor, such as larger areas with blastema-like cells as well as epithelial differentiation, were not evident; on the contrary, atypical anaplastic cells in the stroma and cartilage were present. The tumor was immunohistochemically positive for vimentin. Immunohistochemical studies with CD-99, p53, S-100, Desmin, CD56, and Synaptophysin were negative. The histopathologic diagnosis made then was that of an anaplastic sarcoma of the kidney, at the time a tumor not previously described in the literature.

Not having a specific therapeutic schema for this rare tumor, and after consultation with various clinics in Germany, it was decided to treat the patient with an anaplastic Wilms' tumor Stage II regimen. Cyclophosphamide, Ifosfamid, Carboplatin, and Etoposid, in addition to radiation therapy, were used.

Although there is no general recommendation on the method and timing of investigations for surveillance following surgery for such a rare tumor entity, our follow-up protocol included a bone scan before radiochemotherapy was initiated, followed by a CT of the thorax and of the abdomen 1 month postradiochemotherapy. Six months later (approximately 1 year after the initial diagnosis), a bone scan and CT of the thorax and abdomen were performed once more. Since there was not any clinical or radiological suspicion for recurrence of the disease, the follow-up protocol was switched to surveillance with abdominal ultrasonography every 3 months and CT of the thorax every 6 months for the following 2 years, and then abdominal ultrasonography every 6 months followed by an annual CT of the thorax for the following 5 years to come. The last follow-up was performed in September 2008, with the patient being free of disease and having no significant complications appearing during and after radiochemotherapy.

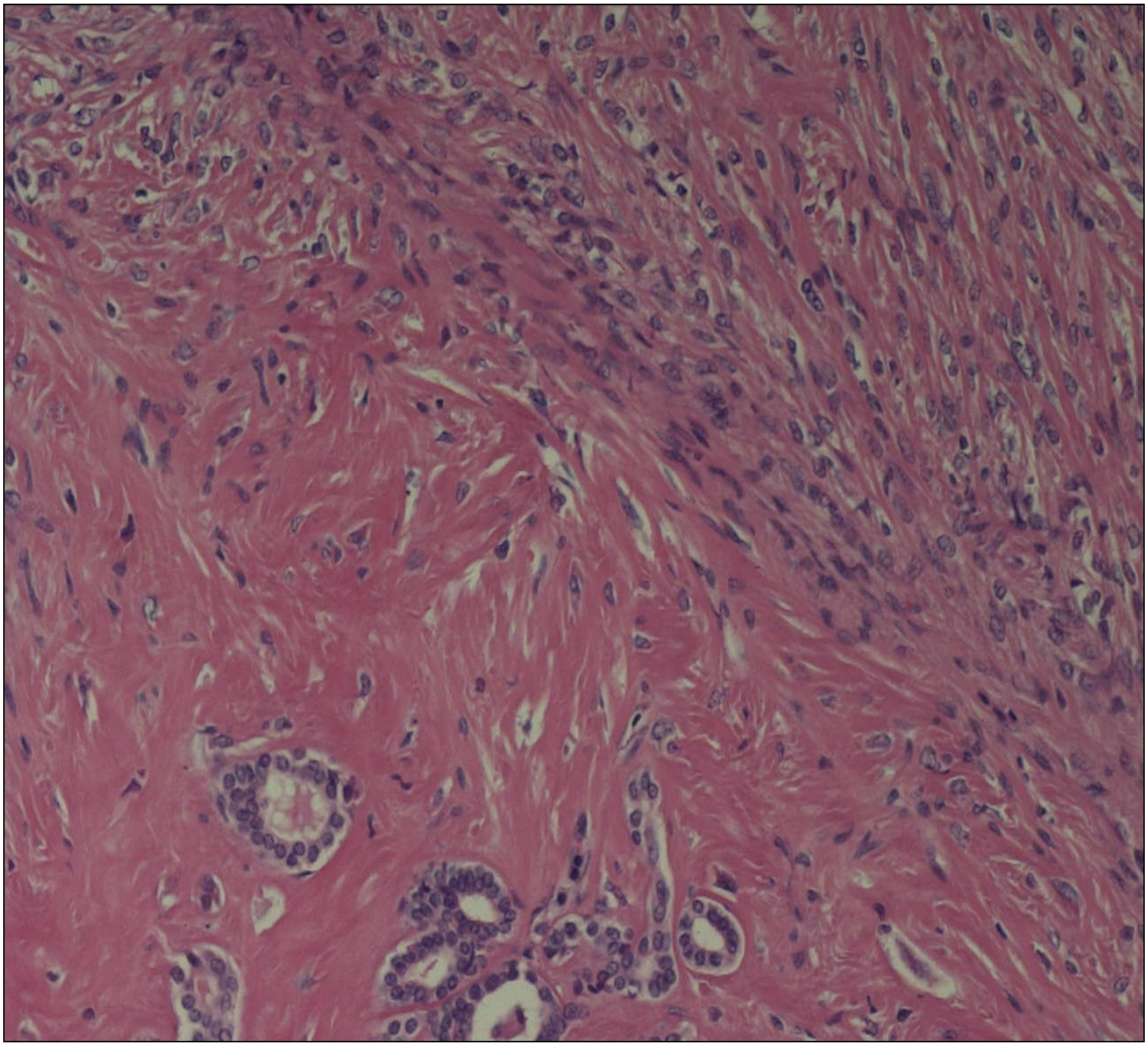

**FIGURE 2:** Histopathologic specimen exhibiting a spindle cell component.

## DISCUSSION

The preoperative diagnosis made for this patient was that of Wilms' tumor or renal cell carcinoma. Wilms' tumor is the most common renal malignancy of childhood. In adults, this disease entity is rare and is usually detected at higher local stages, it exhibits more frequent metastasis, and it develops more toxicity due to therapy. The treatment of Wilms' tumor is one of the great success stories in oncology. Although North America and Europe have different philosophies on preoperative chemotherapy, modern treatment regimens yield overall survivor rates of 90% by both protocols[2]. Treatment in adults is given according to the pediatric protocol, since it is well documented that they exhibit similar overall survival rates[3]. In spite of this success, treatment in patients who exhibit anaplastic or ''unusual'' anaplastic features of this disease is suboptimal.

Vujanić et al.[1], in a novel study, by reviewing sections of tumors coded as ''unusual'' anaplastic Wilms' tumors from the National Wilms' Tumor Study Pathology Center (NWTSPC), the International

Society of Pediatric Oncology (SIOP), and the United Kingdom Children's Cancer Study Group (UKCCSG) trials, recognized a series of 20 cases of a new clinicopathologic entity, the anaplastic sarcoma of the kidney. The three large pathology collections (the NWTSPC, SIOP, and UKCCSG) contained 7400, 4000, and 1600 renal tumors, respectively. The clinical, histopathological, and immunohistochemical features of this tumor are identical with the features of the aforementioned patient. Clinically, it presented as a large renal mass of female predominance and 75% of the patients were under 25 years of age. The histopathologic features exhibited small primitive mesenchymal cells coexisting with a spindle cell component. Anaplastic nuclear changes, exhibiting atypical protean multinucleated cells that contained large anomalous hyperchromatic nuclei and that were dispersed throughout the tumor islands of hyaline cartilage, were also evident. The immunohistochemical features exhibited vimentin-positive staining and p53-negative staining; additionally, negative staining to CD-99, S-100, Desmin, CD56, and Synaptophysin.

The differential diagnosis mostly includes anaplastic Wilms' tumor. One can distinguish between these two entities due to the absence or presence of characteristics consistent with Wilms' tumor. Blastema-like cells, epithelial differentiation, as well as negative immunohistochemical staining of CD56 in the blastema-like foci are absent. Contrarily, atypical anaplastic cells in the stroma and cartilage are present. Other tumors to be considered in the differential diagnosis are renal synovial sarcomas, malignant mesenchymomas, and ectomesenchymomas. Renal synovial sarcomas microscopically are characterized by mitotically active, monomorphic, plump spindle cells with indistinct cell borders growing in short, intersecting fascicles. Grossly identified cysts are lined by mitotically inactive, polygonal eosinophilic cells with apically oriented nuclei[4]. Malignant mesenchymomas are uncommon soft tissue tumors that are composed of two or more different types of malignant mesenchymomal differentiation, in addition to any undifferentiated or fibrosarcomatous elements[5].

Malignant ectomesenchymomas represent a heterogeneous group of tumors, most likely originating from pluripotent primitive neural crest cells. This tumor type is defined as a form that includes ectodermal components represented by neuroblasts or ganglion cells, and differentiated mesenchymal structures of various types. The mesenchymal component is most often a rhabdomyosarcoma[6].

Because the disease was unknown and there was not any available literature of this tumor entity at the time of diagnosis, it was decided to treat the patient as if she had an anaplastic Wilms' tumor Stage II. Adjuvant therapy was therefore performed with Cyclophosphamide, Ifosfamid, Carboplatin, and Etoposid, in addition to radiation therapy.

The patients described by Vujanić et al.[1] were treated according to the initial diagnosis provided by the institutional pathologists and placed on either the NWTSPC or SIOP protocols for treatment. The overall outcome was reasonably good, with 10/13 patients having a median of 8 years of disease survival at follow-up. Similarly, our patient is free of disease and no significant complications have appeared during and after the radiochemotherapy. According to these cases, it is sensible to keep treating these patients as if they had anaplastic Wilms' tumor since they seem to have responded well to this method of treatment.

## CONCLUSIONS

Although it is evident that newer immunohistochemical staining and molecular probes should be applied to this neoplasm in an attempt to learn more about its origin and pathogenesis, it is sensible to keep treating these patients as if they had anaplastic Wilms' tumor, since they seem to have responded well to this method of treatment.
